# Supplementary material for: Improving reconstructions in nanotomography for homogeneous materials via mathematical optimization
Source: Nanoscale Adv. 2024 Jun 3;6(15):3934–47. doi: 10.1039/d3na01089a (PMC11265594; doi:10.1039/d3na01089a)
Supplement: NA-006-D3NA01089A-s001 [file NA-006-D3NA01089A-s001.pdf]

Electronic Supplementary Material (ESI) for Nanoscale Advances.
This journal is © The Royal Society of Chemistry 2024

Electronic Supplementary Information

Improving reconstructions in nanotomography for homogeneous materials via mathematical optimization

Sebastian Kreuz[1], **Benjamin Apeleo Zubiri**[2],
Silvan Englisch[2], **Moritz Buwen**[2],
Sung-Gyu Kang[3,4], **Rajaprakash Ramachandramoorthy**[3],
Erdmann Spiecker[2], **Frauke Liers**[1],
Jan Rolfes[1]

[1]*Department of Data Science, Friedrich-Alexander-Universität Erlangen-Nürnberg, Cauerstr. 11, 91058 Erlangen, Germany*
[2]*Institute of Micro- and Nanostructure Research (IMN) & Center for Nanoanalysis and Electron Microscopy (CENEM), Department of Materials Science and Engineering, Friedrich-Alexander-Universität Erlangen-Nürnberg, Cauerstr. 3, 91058 Erlangen, Germany*
[3]*Department Structure and Nano- / Micromechanics of Materials, Max-Planck-Institut für Eisenforschung GmbH, Max-Planck-Str. 1, 40237 Düsseldorf, Germany*
[4]*Department of Materials Engineering and Convergence Technology, Gyeongsang National University, Jinju-daero 501, 52828 Jinju, Republic of Korea*

May 10, 2024

1 Including the new bounds into SIRT

In SI Figure 1 image reconstructions of the simultaneous iterative reconstruction technique (SIRT) [1,2] are evaluated, comparing SIRT without bounds, SIRT with non-negativity constraints and SIRT with both non-negativity constraints and the newly developed hard upper bounds of this work. The non-negativity constraints are given as

$$f_j \geq 0 \qquad \forall 1 \leq j \leq n, \tag{1}$$

the hard upper bounds as

$$f_j \leq \min_{i \in A_j} \frac{p_i}{R_{ij}} \qquad \forall 1 \leq j \leq n. \tag{2}$$

First, we compare the reconstructions from SIRT without any bounds in SI Figures 1a-1d to the reconstructions from SIRT including the non-negativity constraints in SI Figures 1e-1h. Here, the most streaking artifacts outside the samples are eliminated and the sample edges are much more distinct when including the non-negativity. This is also reflected in the relative mean error (RME)/raw data coverage (RDC) values, where SIRT with included non-negativity bounds performs better than pure SIRT in all cases except for the zeolite reconstruction with 180 projection angles in SI Figures 1c and 1g. In two out of four cases, the bounds could decrease the RME/RDC value by more than 50%.
When additionally including the hard upper bounds in SI Figures 1i-1l, the program reaches lower RME/RDC values in comparison to non-negative SIRT in three out of four cases, being slightly worse for the Cu lattice reconstruction in SI Figure 1l. However, the improvements are not of the same magnitude. The RDC value for the zeolite with 180 projection angles, although better than SIRT with only non-negativity bounds, is still higher than in the reconstruction from pure SIRT.
Visually compared, including the upper bounds into the already existing non-negative SIRT does not induce much improvement. The sample edges seem a little sharper, see, e.g., the edge on the left side of the zeolite in SI Figure 1j compared to SI Figure 1f. Furthermore, a few streaking artifacts outside of the Cu lattice could be eliminated in SI Figure 1l. On the inner part of the samples the new bounds seem to have no notable impact.

2 Varying the TVR-DART parameter λ_{TVR}

In SI Figure 2, the total variation regularized discrete algebraic reconstruction technique (TVR-DART) [3] is evaluated on the zeolite image reconstruction for different weighting parameters λ_{TVR}. One can see that for $10 \leq \lambda_{TVR} \leq 1000$ (SI Figures 2a-2c) the algorithm returns a visually bad reconstruction with strongly varying material density. In these cases, the algorithm does not converge to a stopping criterion in the given 250 iterations. Note that the visual improvement gained by increasing the number of iterations is negligibly small. When increasing λ_{TVR} to 1150, the algorithm suddenly converges. The visual improvement in reconstructions from $\lambda_{TVR} = 1000$ to $\lambda_{TVR} = 1150$ (SI Figure 2c to SI Figure 2d) is immense, in the latter, the material seems to have the desired homogeneous density. Increasing λ_{TVR} further up to 10000 does not change the conversion rate and also the visual results do not differ by much, compare e.g. SI Figures 2d and 2f. Thus, there appears to be a specific turning point in parameter λ_{TVR} for TVR-DART, that may improve its performance drastically. Contrary to this, the RDC values are monotonically increasing in the reconstructions with increasing λ_{TVR} in SI Figure 2, even though the reconstructions with larger λ_{TVR} are visually by far the better reconstructions. This supposed contradiction is further investigated in SI Figure 3 by comparing the image reconstruction of a simulated sample to its original.

In SI Figure 3a, with a smaller parameter $\lambda_{TVR} = 10$, the sample interior exhibits random black spots, called pepper-noise. The homogeneity over the sample is therefore disturbed, and also the edges are very unclear. The reconstruction reaches a relative mean error of 0.0472. When increasing the parameter to $\lambda_{TVR} = 500$ in SI Figure 3c, the pepper-noise vanishes and the edges are carved out a little better. However, the relative mean error of this reconstruction is 0.1115. Even though it is the visually better reconstruction, the error is more than twice as high. Subtracting from both reconstructions the original simulated sample pixel by pixel, SI Figures 3b and 3d emerge. For the smaller parameter, the empty background outside the sample still contains a lot of artifacts that vanish for the larger parameter. Inside the sample in SI Figure 3b, the pepper noise and some small density deviations become visible. Inside the sample in SI Figure 3d, these deviations appear larger, and especially the edges of the sample exhibit sharp deviations against the original. Looking at the density values of the solution, it seems the larger penalization parameter leads to a lower material density estimation variable ρ_2 ($G = 2$) (see TVR-DART description) than in the smaller penalization parameter case. This means the overall image gray values are pressed down by the penalization. This effect can be seen by the overall darker gray values inside the sample in SI Figure 3d compared to SI Figure 3b. This is causing a higher relative mean error. When looking at the difference of the reconstruction from CSHM to the original in SI Figure 3f, one can see that the homogeneity of the sample is much better than in SI Figure 3d, only near the edges of the sample the CSHM reconstruction deviates from the original.

3 Local thickness maps and pore size distributions

SI Figure 4 shows the calculated local thickness map (LTM) and pore size distribution (PSD) of the SIRT reconstruction with positivity constraint and 180 projections of the porous zeolite particle from Figure 4b. We use this pore size distribution as 'ground truth' for the comparison of the following LTMs and PSDs and will refer to it as 'SIRT 180'.
In SI Figure 5, the PSD of CSHM (20 projections) covers all main peaks as the PSD of SIRT 180, but with a systematic underestimation of 10-20 nm for pores larger than 100 nm, whereas pores < 100 nm are represented very well. In contrast, the other techniques cover larger pores up to 275 nm, but partly fail in the detection of other main peaks compared to SIRT 180. The underestimation in the CSHM PSD can be explained by some falsely re-

constructed material of lower intensity adjacent to the pore walls, which is assigned to material instead of pore by the applied Otsu thresholding method. This erroneously reconstructed material is also visible in the other reconstructed slices, but rather assigned to pore space when thresholding. As already mentioned in the main manuscript, the soft upper bounds of CSHM penalize bright spots that show bigger densities than the main material density and push the exceeding material to other pixels. This explains the additional intensities in the inside of pores for CSHM compared, e.g., to CS. By using machine learning segmentation methods, this discrepancy could easily be taken care of, since the main intensity of the zeolite material is clearly discernible in CSHM.

Similarly to the results using 20 projections, the PSD in SI Figure 6 of CSHM using 30 projections covers all main peaks as SIRT 180, but again with a systematic underestimation (even though reduced compared to 20 projections) for pores larger than 100 nm, whereas pores $<$ 100 nm are covered very well. In contrast, the other techniques again cover larger pores up to 275 nm, but partly fail in the detection of other main peaks compared to SIRT 180. As expected, the local thickness maps of all reconstructions look more similar to SIRT 180 when reconstructed with 30 projections compared to those with only 20 projections.

The LTM of the CSHM reconstruction including a missing wedge in SI Figure 7 shows by far the least merging of pores compared to all other methods. This corresponds to less and more pronounced peaks in the respective pore size distribution of CSHM, even though the general pore size underestimation for pores larger than 100 nm is also present similar to the reconstructions without missing wedge. However, the pores at $\sim$140 nm and $\sim$170 nm for CSHM are in a better agreement with SIRT 180 compared to the other techniques.

When comparing the different SIRT reconstructions in SI Figure 8, then it is apparent that SIRT without bounds and 20 projections exhibits the worst artifacts and the pores are not well reconstructed, which is further reflected in the LTM and PSD. In contrast, SIRT non-negative and bounded using 20 projections lead to very similarly looking results for both LTMs and PSDs, and are already quite close to SIRT 180, especially when compared to SIRT without bounds. All three SIRT reconstructions using 180 projections (without bounds, bounded and non-negative) lead to very similar results, except for a slight shift to a lower value for the PSD peak at $\sim$175 nm of the reconstruction without bounds.

The PSDs of TVR-DART $\lambda_{TVR} = 10$ and $\lambda_{TVR} = 1000$ in SI Figure 9 exhibit a larger fraction and broader distribution of smaller pores below 150 nm compared to the reconstructions using higher λ_{TVR}. However, especially the peak at $\sim$220 nm matches the SIRT 180 PSD better than the TVR-DART PSDs with larger λ_{TVR} values. When comparing the local thickness maps, it becomes apparent that lower λ_{TVR} values (especially for $\lambda_{TVR} =$ 10) lead to locally wrong pore reconstruction and recognition in the respective LTM, especially on the outside surface of the particle.

4 Animated videos of the tilt series

SI Video 1 is showing an animation of the full tilt series of projections of the experimental electron tomography data of a macroporous zeolite particle on the plateau of a tomography tip as shown in Figure 2 of the main manuscript.

SI Video 2 is showing an animation of the full tilt series of projections of the experimental absorption-contrast nano-CT data set of a copper microlattice structure as shown in Figure 3 of the main manuscript.

References

1 P. Gilbert, *Journal of theoretical biology*, 1972, **36**, 105–117.

2 A. C. Kak and M. Slaney, *Principles of computerized tomographic imaging*, SIAM, 2001.

3 X. Zhuge, W. J. Palenstijn and K. J. Batenburg, *IEEE Transactions on Image Processing*, 2015, **25**, 455–468.

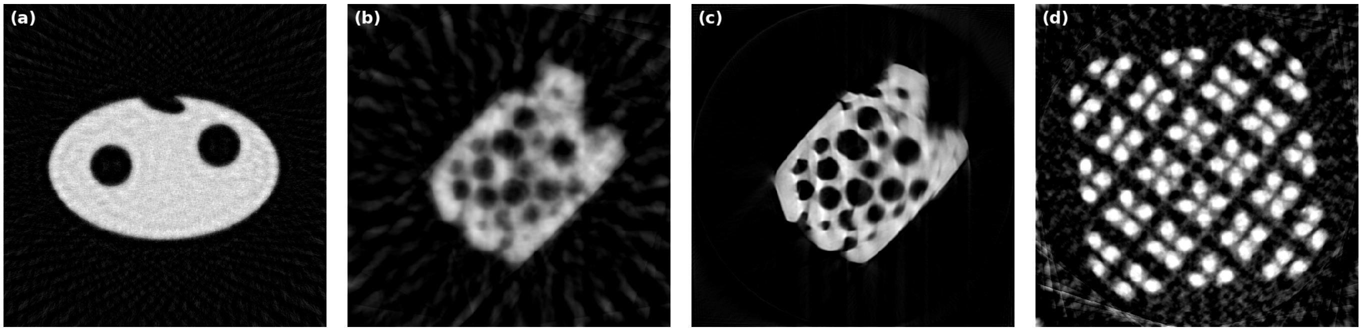

(a) SIRT, 45 angles, RME: 0.2454 **(b)** SIRT, 20 angles, RDC: 18.0740 **(c)** SIRT, 180 angles, RDC: 4.3590 **(d)** SIRT, 40 angles, RDC: 32.4345

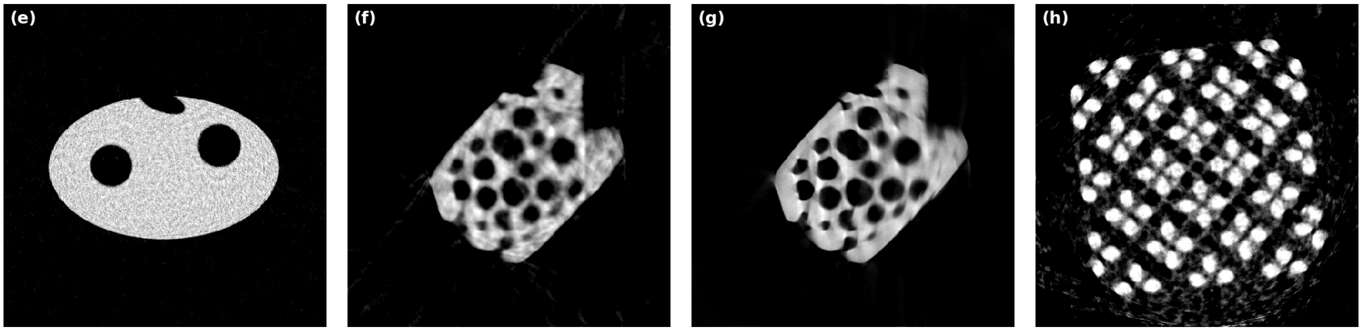

(e) SIRT non-negative, 45 angles, RME: 0.1190 **(f)** SIRT non-negative, 20 angles, RDC: 8.3933 **(g)** SIRT non-negative, 180 angles, RDC: 6.3723 **(h)** SIRT non-negative, 40 angles, RDC: 32.0757

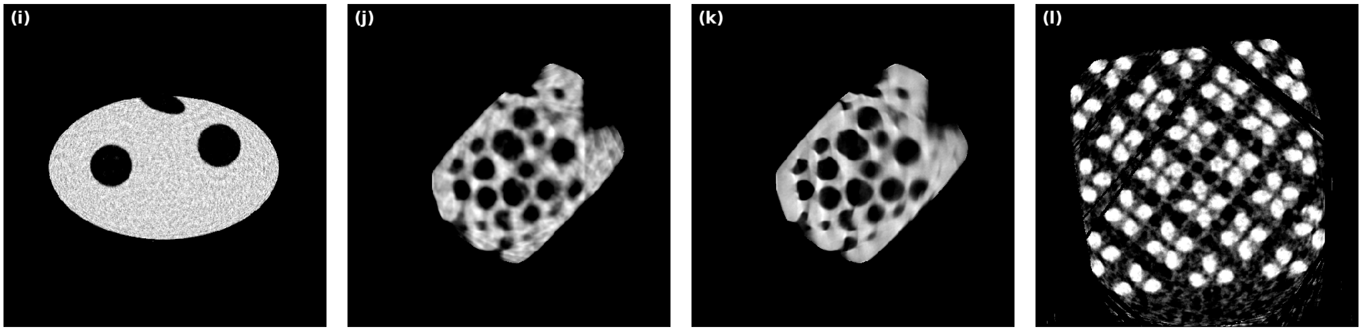

(i) SIRT bounded, 45 angles, RME: 0.1165 **(j)** SIRT bounded, 20 angles, RDC: 7.5220 **(k)** SIRT bounded, 180 angles, RDC: 5.9427 **(l)** SIRT bounded, 40 angles, RDC: 32.1840

SI Figure 1 Comparison of SIRT without bounds (a)-(d) to SIRT with non-negativity constraints (called SIRT non-negative) (e)-(h) and SIRT with non-negativity and hard upper bound constraints, (in the following called SIRT bounded) (i)-(l)

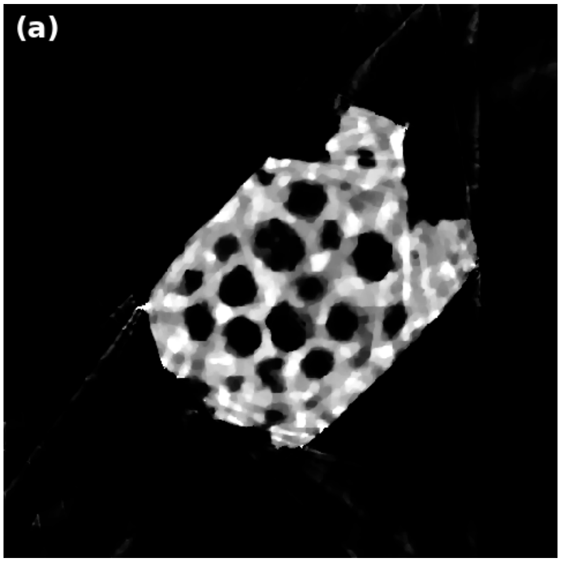

(a) $\lambda_{TVR} = 10$, RDC: 11.3882

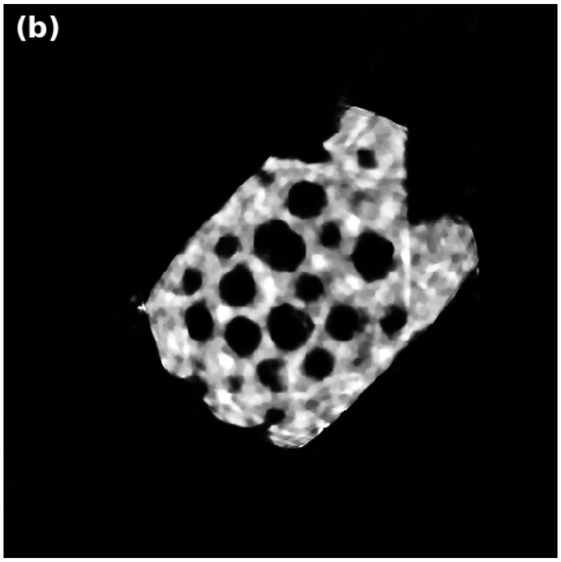

(b) $\lambda_{TVR} = 100$, RDC: 11.4662

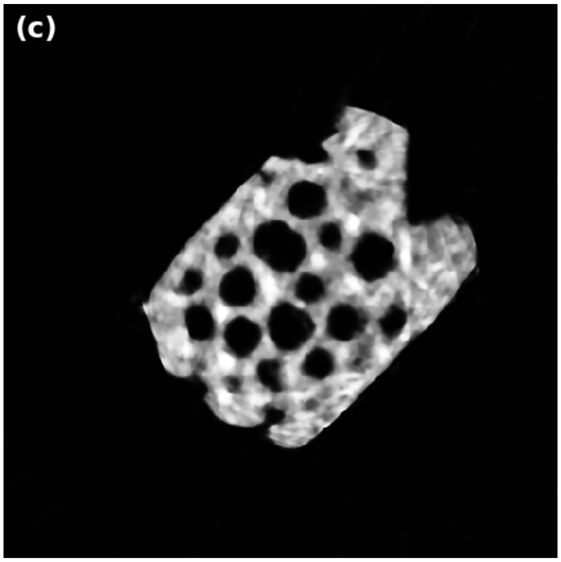

(c) $\lambda_{TVR} = 1000$, RDC: 12.6422

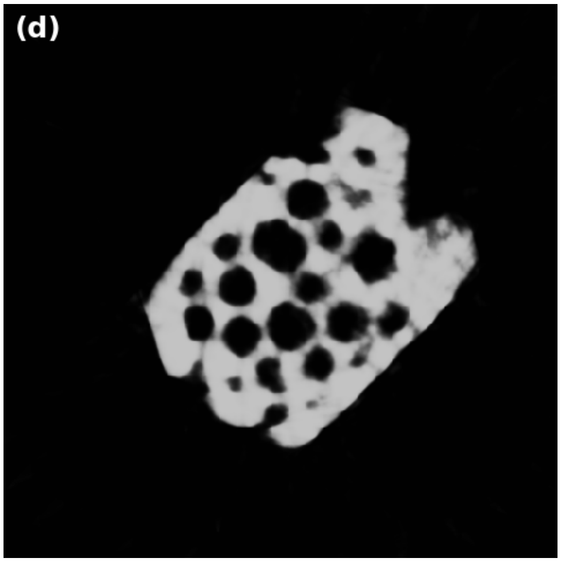

(d) $\lambda_{TVR} = 1150$, RDC: 13.2136

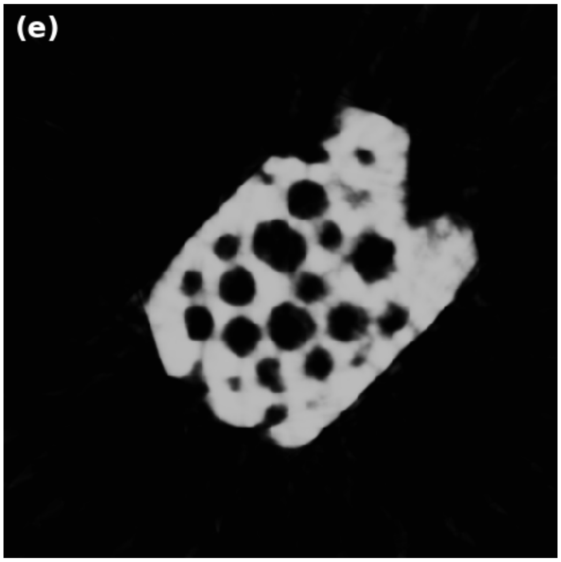

(e) $\lambda_{TVR} = 5000$, RDC: 19.1119

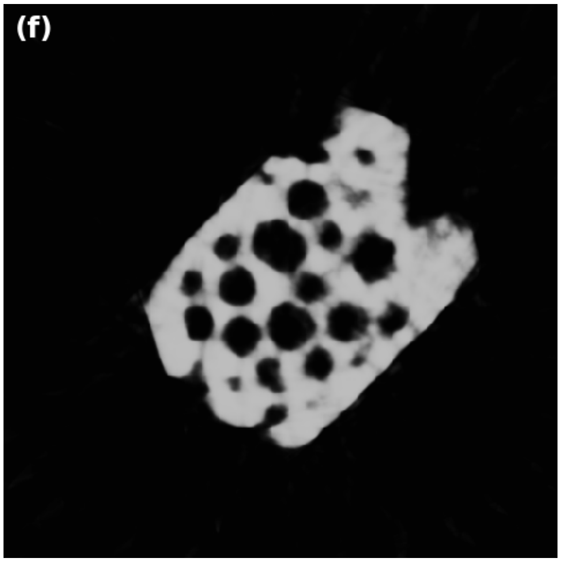

(f) $\lambda_{TVR} = 10000$, RDC: 19.2447

SI Figure 2 TVR-DART comparison reconstructions with 20 projection angles and 512^2 pixels for different parameters λ_{TVR}

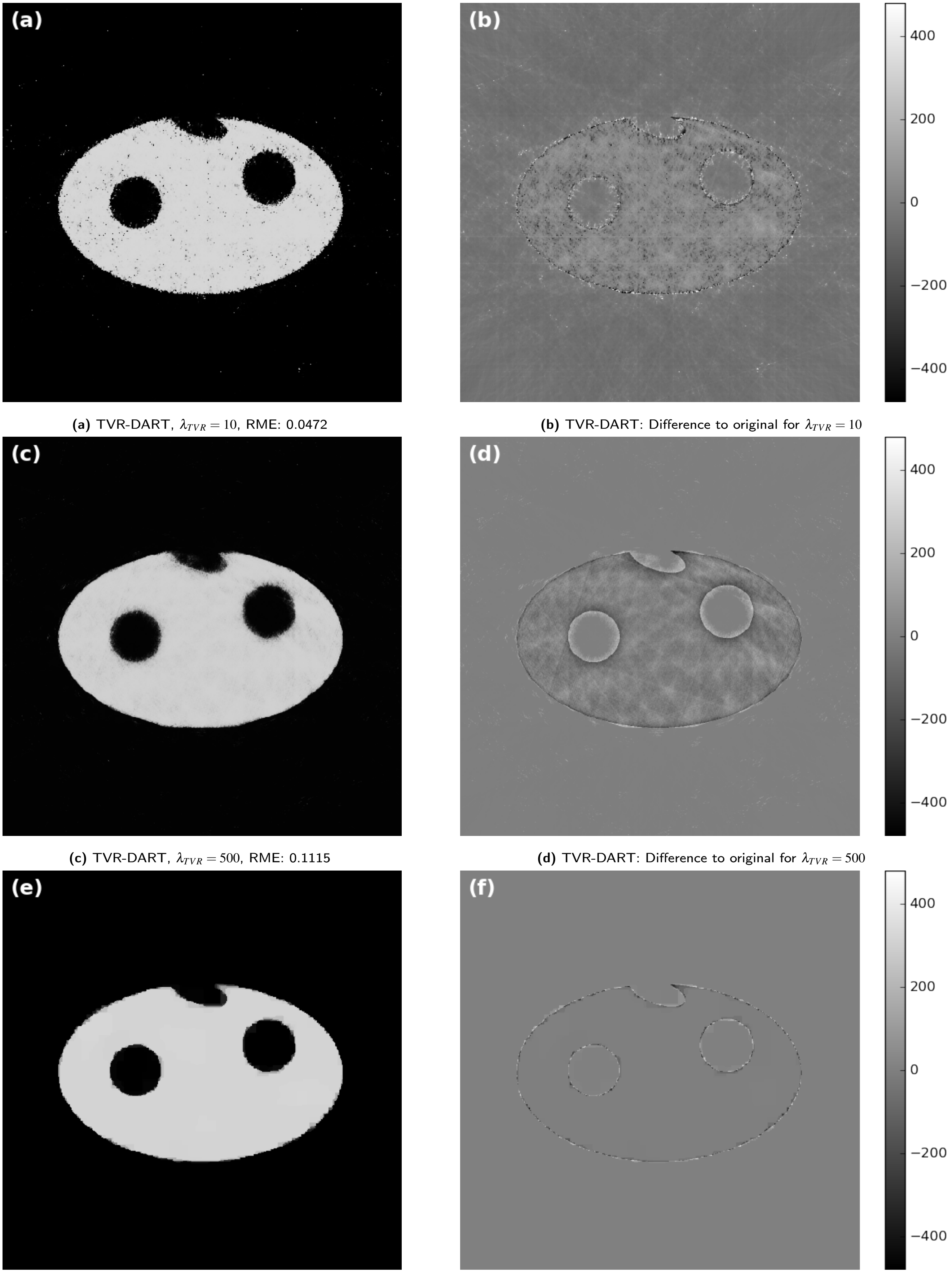

(a) TVR-DART, $\lambda_{TVR} = 10$, RME: 0.0472

(b) TVR-DART: Difference to original for $\lambda_{TVR} = 10$

(c) TVR-DART, $\lambda_{TVR} = 500$, RME: 0.1115

(d) TVR-DART: Difference to original for $\lambda_{TVR} = 500$

(e) CSHM, RME: 0.0223

(f) CSHM: Difference to original

SI Figure 3 Image reconstructions with 10 projection angles and 512^2 pixels on a simulated sample, and their pixel by pixel differences to the original. Compared are TVR-DART with $\lambda_{TVR} \in \{10, 500\}$ and CSHM with $\lambda_{CS} = 20000$.

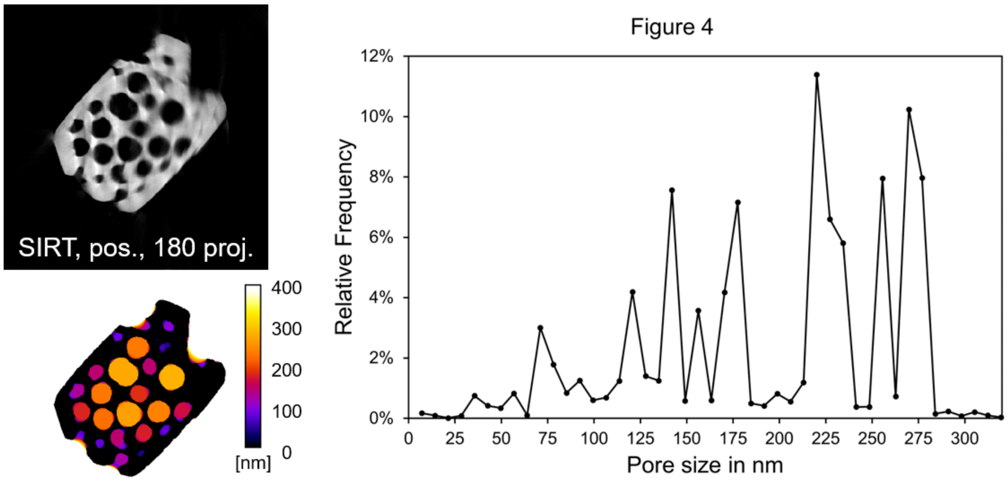

SI Figure 4 (top left) SIRT reconstruction with positivity constraint and 180 projections of the porous zeolite particle from Figure 4b, (bottom left) its corresponding local thickness map, and (right) pore size distribution.

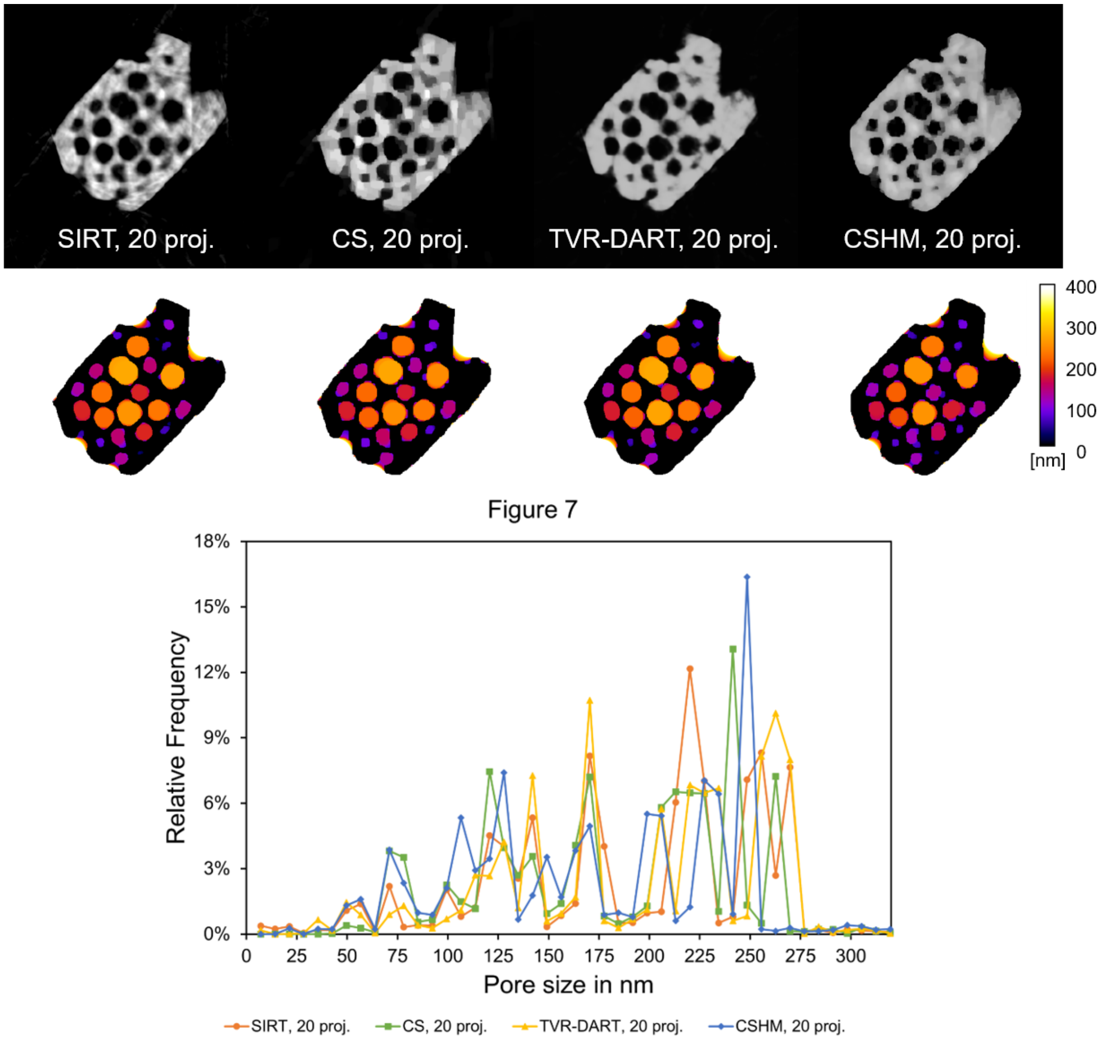

SI Figure 5 (top row) Different reconstructed slices of the porous zeolite particle using 20 projections from Figure 7, (middle row) their corresponding local thickness maps, and (bottom) pore size distributions.

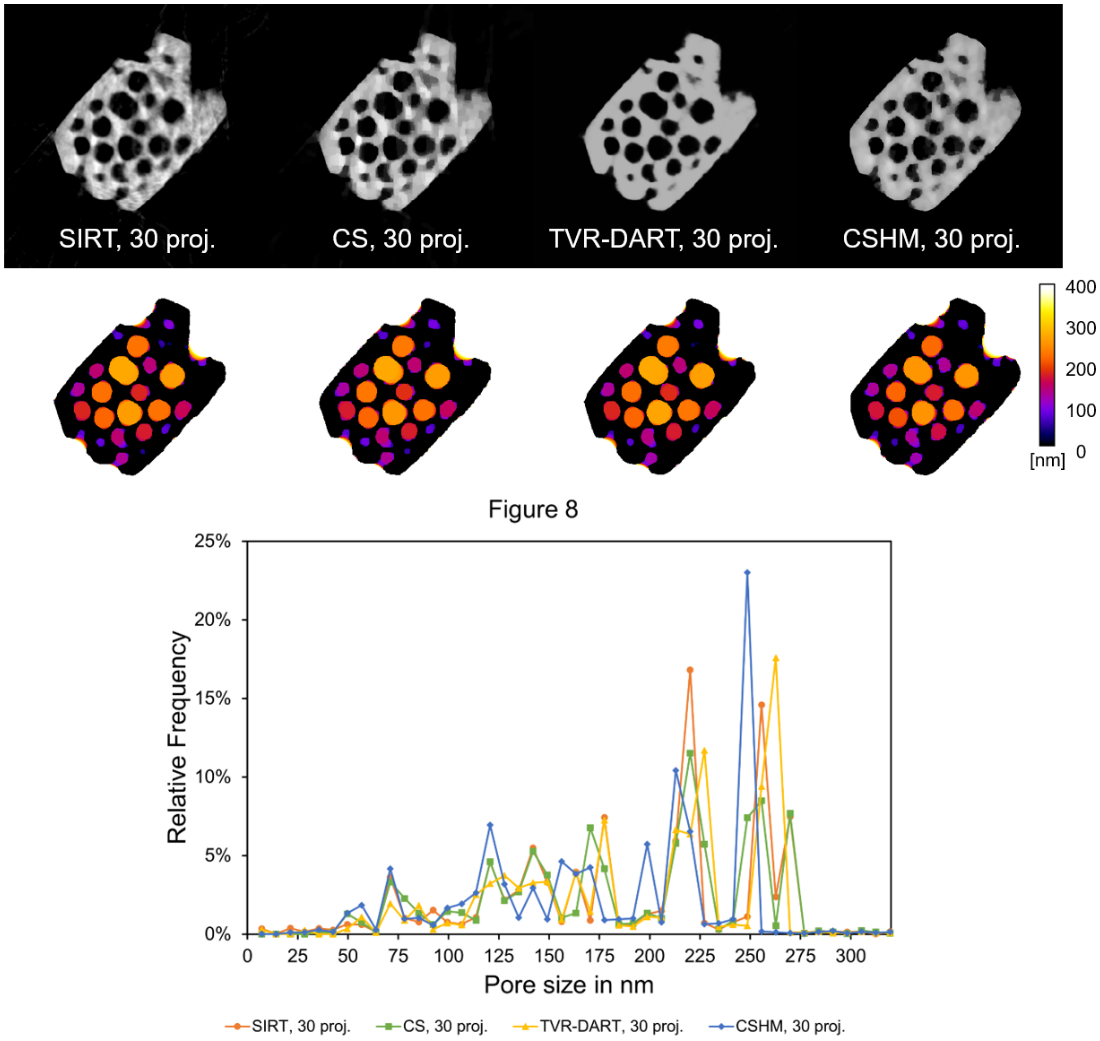

SI Figure 6 (top row) Different reconstructed slices of the porous zeolite particle using 30 projections from Figure 8, (middle row) their corresponding local thickness maps, and (bottom) pore size distributions.

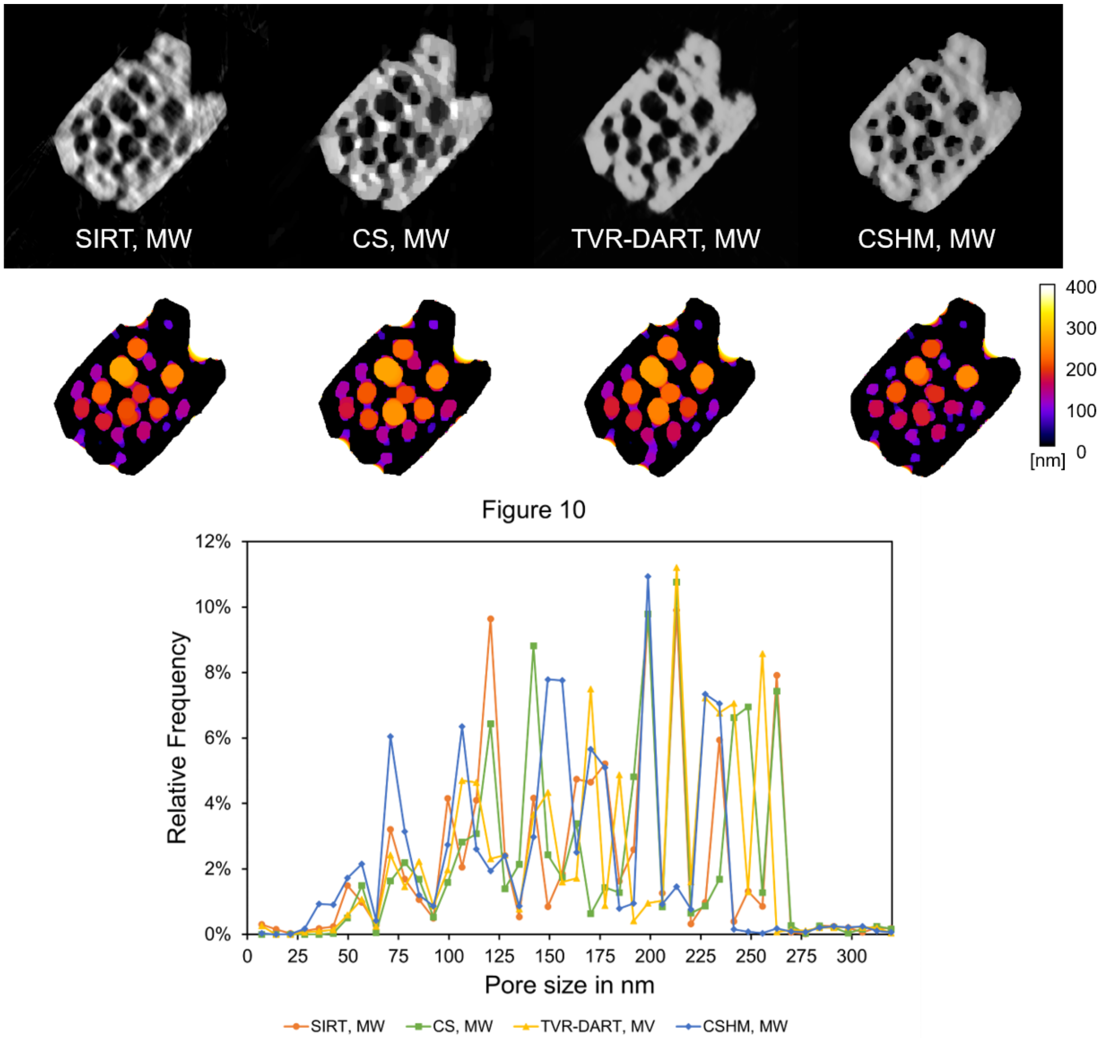

SI Figure 7 (top row) Different reconstructed slices of the porous zeolite particle with missing wedge from Figure 10e-h, (middle row) their corresponding local thickness maps, and (bottom) pore size distributions.

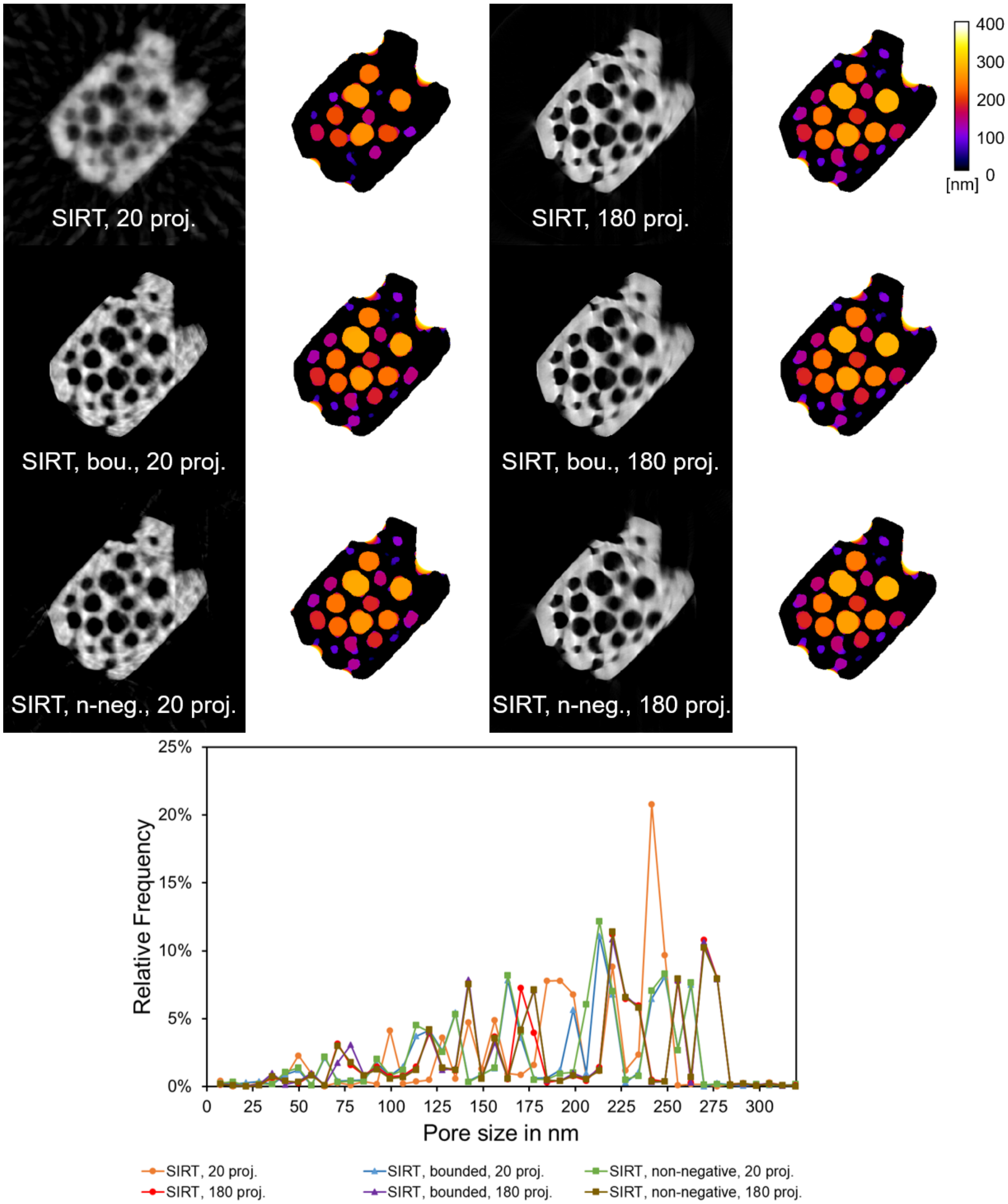

SI Figure 8 Different SIRT reconstructions (20 and 180 projections, each without bounds, bounded and non-negative) of the porous zeolite particle from SI Figure 1, their corresponding local thickness maps, and pore size distributions.

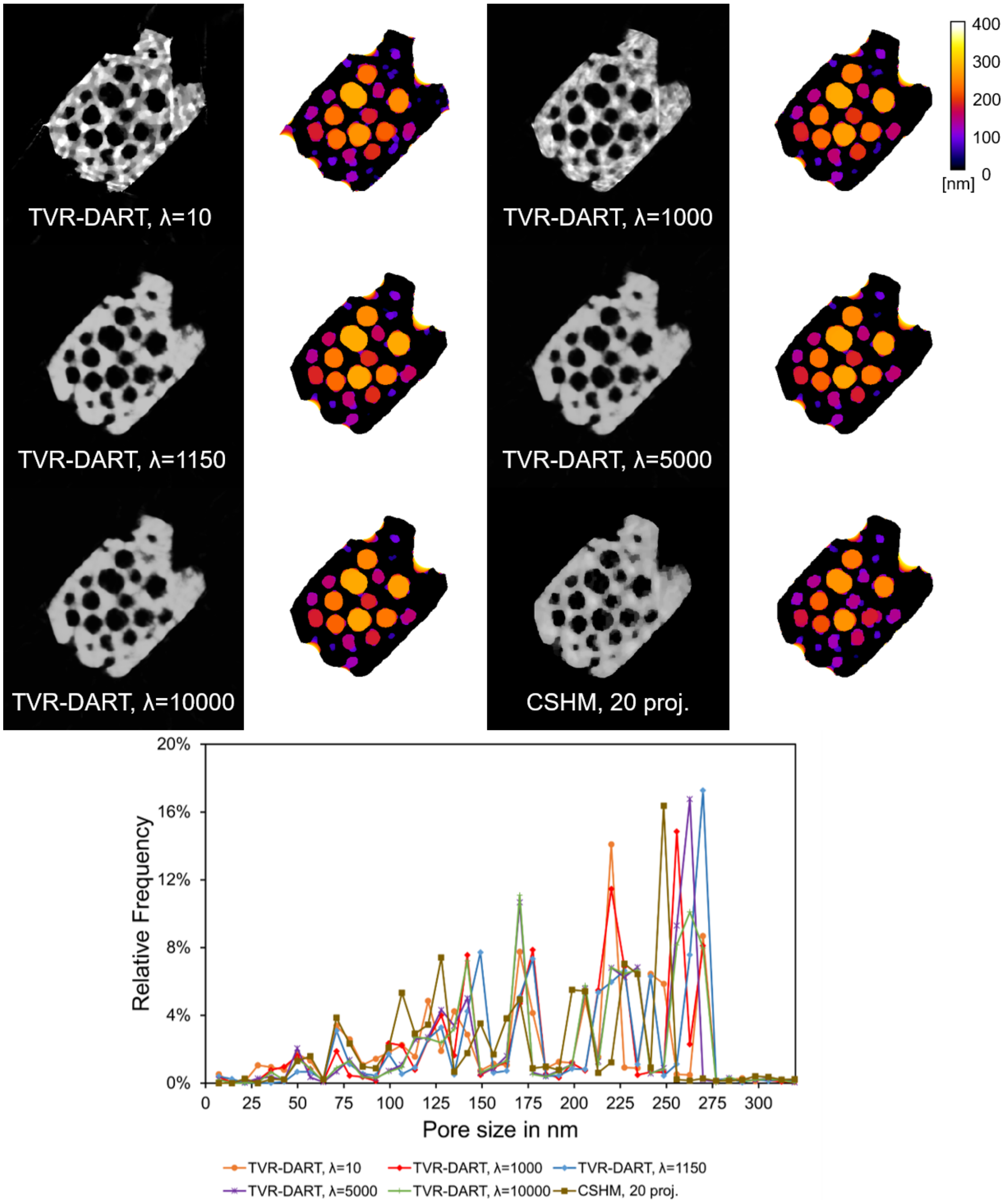

SI Figure 9 TVR-DART reconstructions for different parameters λ_{TVR} and CSHM slice (20 projections) of the porous zeolite particle from SI Figure 2, their corresponding local thickness maps, and pore size distributions.
